# Improving health information system for malaria program management: Malaria Frontline Project lessons learned from Kano and Zamfara States, Nigeria, 2016-2019

**DOI:** 10.11604/pamj.2023.46.17.40921

**Published:** 2023-09-12

**Authors:** Adefisoye Adewole, Olufemi Ajumobi, Ndadilnasiya Waziri, Amina Umar, Usaini Bala, Saheed Gidado, Patrick Nguku, Perpetua Uhomoibhi, Basheer Muhammad, Munira Ismail, Shelby Cash, John Williamson, Stephen Patrick Kachur, Peter McElroy, Kwame Asamoa

**Affiliations:** 1 African Field Epidemiology Network, Nigeria Country Office, Abuja, Nigeria,; 2 School of Public Health, University of Nevada, Reno, Reno, Nevada,; 3 National Malaria Elimination Program, Federal Ministry of Health, Abuja, Nigeria,; 4 Kano State Ministry of Health, State Malaria Elimination Program, Kano, Nigeria,; 5 Zamfara State Ministry of Health, State Malaria Elimination Program, Gusau, Nigeria,; 6 Malaria Branch, Division of Parasitic Diseases and Malaria, Center for Global Health, Centers for Disease Control and Prevention,; 7 Mailman School of Public Health, Columbia University Medical Center, New York, New York, United States of America

**Keywords:** Lessons learned, Malaria Frontline Project, data quality improvement, Zamfara State, Kano State

## Abstract

The U.S. Centers for Disease Control and Prevention in collaboration with the National Malaria Elimination Program and the African Field Epidemiology Network established the Malaria Frontline Project to provide innovative approaches to improve the malaria program implementation in Kano and Zamfara States, Nigeria. Innovative approaches such as malaria bulletin, malaria monitoring wall chart, conduct of ward level data validation meetings and malaria dashboard have helped improve the use of data for decision making at all levels. Innovative approaches deployed during the project implementation facilitated data analysis and a better understanding of malaria program performance and data utilization for decision making at all levels. These innovative approaches may improve malaria control program performance in Nigeria and other resource limited countries.

## Project evaluation

Accurate and reliable data are critical for effective planning, coordination, and delivery of health services. Routine health management information systems (HMIS) provide essential data for decision-making process to optimize service delivery and improve program performance [1]. Public health program decision-making depends greatly on the timely availability of good data. Effective use of HMIS data for decision making depends in part, on the quality of data [1]. Challenges and opportunities with data recording and reporting processes begin at the health facility level. Suboptimal data quality is a major problem in Nigeria´s HMIS [2], ranging from data incompleteness, entry errors, underreporting, and data discrepancies between patient registers and aggregate data summaries. Improving the quality of routine data is a priority given the potential of routine data to contribute to effective program monitoring and performance management. Periodic data quality review with feedback has been shown to improve completeness, timeliness, and accuracy of facility reporting. The Malaria Frontline Project (MFP), a CDC project implemented in Kano and Zamfara States to support the Nigeria National Malaria Elimination Program (NMEP) improved the implementation of malaria interventions recommended by World Health Organization (WHO) and supported by global donors and partners [3]. MFP was implemented through the National Stop Transmission Program (NSTOP) of the African Field Epidemiology Network (AFENET) Nigeria Country office in 20 of the 44 Local Government Areas (LGAs) in Kano State and all the 14 LGAs in Zamfara State. The goal of the project was to strengthen technical capacity of healthcare workers (HCWs) to implement malaria interventions and improve malaria surveillance in the project states. Suboptimal data quality was identified at health facility and LGA levels prior to MFP implementation in both States. Historically, HCWs focused mainly on meeting timelines for data reporting to the LGA level, with less focus on assessing quality of the data originating from health facilities. Most often health facility managers did not analyze and use the data they generate for decision making. Previously, LGA Monitoring and Evaluation (M&E) officers collected and entered all health facility data into District Health Information System-version 2 (DHIS-2) without adequate on-site validation of the data, resulting in many discrepancies subsequently observed during data quality audit at the national and sub-national levels. To achieve the MFP objective of strengthening malaria surveillance through higher quality malaria data recording, reporting, and analysis for decision making, several strategies were implemented to address identified problems. This paper documents the contributions of the MFP to improve malaria surveillance and service delivery at the health facility and LGA levels in the project areas.

**MFP contributions to improve data quality, analysis, and use of the results for program implementation in Kano and Zamfara States. Capacity building of HCWs in data analysis:** the MFP adapted the NSTOP three-prong strategy of 1) classroom didactic training, 2) experiential group work including post-training assignments and case studies, and 3) field visit training to perform data-driven health facility supportive supervisory visits. The strategy improved the capacity of HCWs in basic data analysis using modular trainings in malaria surveillance and data management. This enabled HCWs at health facility and LGA levels to conduct basic data analysis of selected indicators and interpretation of the results to the identified challenges affecting the program. The DHIS2 platform has 26 malaria indicators for health workers to report. Together with health facility and LGA leadership, six indicators [reporting rate, reporting timeliness, access to malaria testing, case management, intermittent preventive treatment in pregnancy, and access to long lasting insecticidal nets (LLIN)] whose analysis reflect program performance were selected for analysis and tracking of program performance at the health facility and LGA levels.

**Ward level data validation to improve data quality:** prior to MFP, the United States President´s Malaria Initiative (PMI) funded partner, Malaria Action Program for States (MAP) supported the Zamfara State malaria program to conduct monthly data validation at the LGA level for MAP supported health facilities. Health facility officers converged at the LGA headquarters for two to three days´ monthly data validation meeting with technical support from the MAP data officer. The MAP project supported selected facilities in an LGA, thus data quality for the whole LGA was diluted by low quality data from the non-MAP supported facilities. The MAP project ended just before the MFP commenced in Zamfara State. The MFP supported all health facilities in an LGA to participate in monthly data validation meetings where data were reconciled from the facility register with the monthly summary form. This followed the MAP format, and the support covered all 14 LGAs in Zamfara State and the health facilities in the 20 project LGAs in Kano State. However, after two consecutive monthly data validation meetings, it was evident the approach was not sustainable because of the enormous financial commitment, time, and technical constraints. The MFP decentralized the meetings to ward level. An LGA has between eight to fifteen wards, and most wards have three to five facilities except where the ward is in the state capital where there are more facilities. The ward level meetings involved fewer facilities, shorter meeting duration (two to three hours), shorter travel distance from facility to meeting place (<2km based on revised “Reaching Every Ward” strategy) [4], and no cost incurred by the program for transport or per diem for staff. Importantly, patients could still have access to care on the data validation meeting day even at facilities staffed by only one person. This did not happen before the innovation of ward-level data validation and has helped ensure sustainability of the intervention after the project.

**Figure 1:**
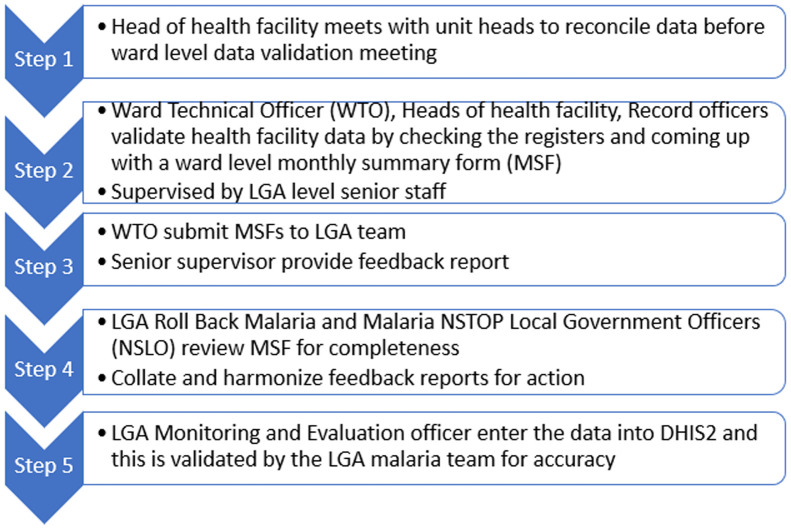
activity and data flow from Ward Level Data Validation (WLDV) to DHIS2 data entry

**Figure 2:**
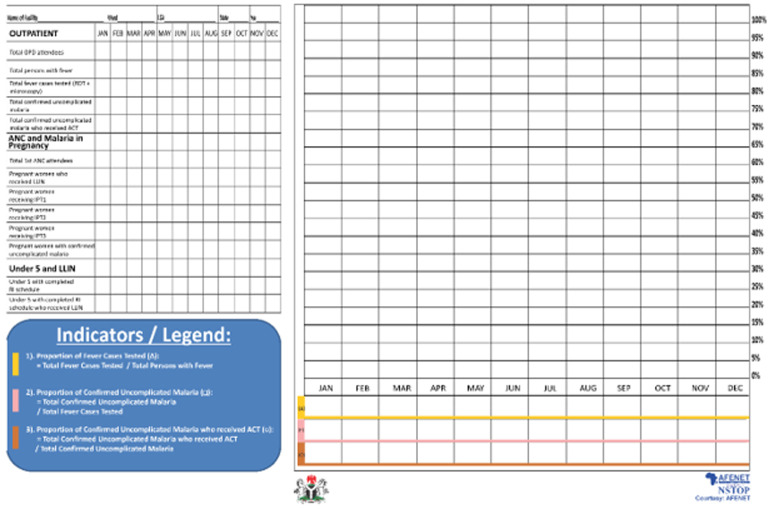
malaria monitoring wall chart

The validated facility data from the ward was submitted to the LGA Monitoring & Evaluation (M&E) officer after collation by the LGA Malaria Focal Person. The LGA M&E officer collated all the ward data and entered it into the DHIS2 platform. The MFP technical officers at LGA known as NSTOP Local Government officers provided technical support to the facilities, wards, and the LGA team. Data validation at facility and ward levels improved data quality (Figure 1), and the corresponding analyses projected on the malaria monitoring wall chart were used for program monitoring and decision making (Figure 2). This decentralization of data validation to the ward level was a novel approach to address quality assurance of public health surveillance in Nigeria.

**Development and use of malaria monitoring wall chart to enhance decision making:** the Malaria Monitoring Wall Chart was developed by MFP to increase data visibility by publicly displaying the selected six performance indicators on the chart at each health facility, that is, the service delivery level, and on the LGA noticeboard. This chart was adapted from MAP and WHO´s Road to Health Chart. The chart helps staff, managers, and visitors to visually track the malaria program performance. By the end of the project period, all health facilities, wards, and LGAs had malaria monitoring wall charts displayed. The chart also helps visualize disease trends, adherence to national diagnosis and treatment guidelines, uptake of intermittent preventive treatment of malaria during pregnancy (IPTp), LLIN uptake among pregnant women and children under five years (Figure 2). This innovation brought a paradigm shift for HCWs: from being data collectors and reporters, to becoming managers of higher quality data and its use for decision making. After each monthly ward-level data validation meeting, the officer in charge at the health facility level enters the validated data (another innovation of the project) into the malaria monitoring wall chart. The performance of the health facility is tracked based on the selected key malaria indicators on the monitoring wall chart and use of the analyzed data for decision making. The MFP printed and distributed malaria monitoring wall charts across 726 and 739 supported health facilities in Kano and Zamfara States, respectively, from 2017 [5]. Since 2020, Kano State government took ownership of the printing and distribution of the malaria monitoring wall charts across Kano State health facilities.

**Figure 3:**
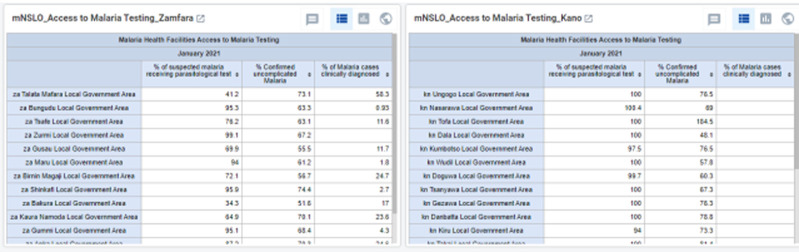
DHIS2 data describing the distribution of access to malaria testing by LGAs in Kano and Zamfara States

**AFENET Malaria DHIS2 dashboard:** the MFP also adapted the DHIS2 routine immunization dashboard module for malaria [6]. The malaria DHIS2 dashboard draws data from DHIS2 platform and presents it in an easy-to-visualize format in excel. It is based on the analysis of selected malaria program performance indicators which state and LGA officers have access to, for viewing program performance and decision-making (e.g., when/where to perform HF supportive supervision visits). These indicators include reporting rate, reporting timeliness, access to malaria testing, case management, intermittent preventive treatment in pregnancy and access to LLIN. Each indicator helps track program performance at the LGA and state levels. Based on agreed cut-off points and color codes (defined by the National Malaria Elimination Program) good performance (green color), medium performance (yellow color), and poor performance (red color) are indicated. The dashboard affords the LGA and state teams the opportunity to examine the data critically and identify good and poor performing health facilities. Health facilities with demonstrated performance challenges indicative of yellow and red codes are prioritized for supportive supervision visits (Figure 3).

The LGA and state officers review the dashboard monthly. Based on the LGA and state performance of the selected malaria indicators, they identify specific LGA, wards, and health facilities with poor performance. The health facility supportive supervision visits were conducted with the aim to address challenges at poor performing health facilities and LGAs, respectively. On-the-job training and mentoring of health facility staff were conducted by the LGA supervisory team. Some well-performing health facilities were also selected at random for visits and to verify their data on the DHIS2 dashboard. The excel-based dashboard developed with the Malaria Data Management Expert group (comprising Nigeria National Malaria Elimination Program and development partners) was initially used as a performance monitoring tool. It has both national and state components. The national dashboard shows the six selected key indicators for both project states using the agreed threshold for each indicator and corresponding color codes to delineate good, medium, and poor performance.

**Figure 4:**
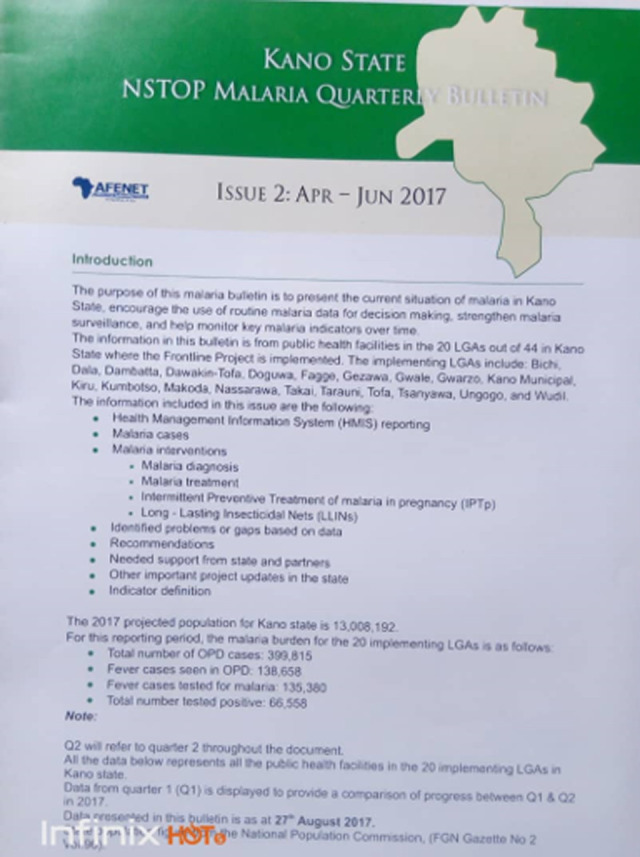
malaria quarterly bulletin

**State malaria bulletin to facilitate decision making by authorities:** state Malaria Bulletin was established in each project state to make data and program performance visible to health authorities at many levels, health partners, and the public. The bulletin is the first of its kind for any disease program in the country providing quarterly and annual reports that reflect malaria control program performance indicators. The bulletin was written in plain English and targeted policymakers and politicians. They are not technical experts and do not have the time to read several pages of document, but they are very influential and make decisions on funding. Data visualization in the bulletin form is the best approach for this audience. The bulletin had few selected indicators from the facility level and an appendix of concise operational definitions of terms to aid further understanding. It is envisioned that in future, other public health programs will have selected performance indicators to report which may be added to the quarterly bulletin (Figure 4).

**Health facility service delivery assessment:** during the MFP implementation, there were inconsistencies between observed and expected number of health facilities submitting monthly reports in the project areas. To ensure an accurate number of expected facilities to submit reports, the project conducted a health facility listing where geo-coordinates of each facility, category of facility, type of services provided, facilities reporting to DHIS2 platform (reasons for non-reporting), and number and category of facility staff were documented (23). This provided an accurate number of facilities expected to submit a monthly report, and therefore, the catchment area denominator needed to calculate rates. Recording the staff numbers and categories at facility showed that less than 10% of PHCs in Zamfara and 20% of PHCs in Kano State had the required minimum staff established by the Ministry of Health to deliver the expected service [5].

The MFP strategies improved data quality and use in Kano and Zamfara States by building capacity of HCWs in basic data analysis and the use of the results to improve program performance. The State Malaria Bulletin provided a brief update about the program performance indicators. The data harmonization and review at the ward level provided timely feedback on key malaria indicators to health authorities at the LGA level across the MFP States. The MFP adapted a NSTOP polio program strategy to build capacity of healthcare workers in malaria surveillance, data management and analysis, and the use of data for decision making especially at facility, LGA and state levels. The MFP contributions in Kano and Zamfara States addressed gaps associated with poor data quality and improved the use of data for decision making by program managers at state, LGA and HF levels. The selection and use of six performance indicators for routine monitoring improved malaria program management at facility and LGA levels. Studies assessing the quality of routine HMIS data have shown persistent challenges especially at the health facility level where these data originate from [7]. The decentralization of the validation meeting to ward level offered an important and sustainable management strategy for Ward and LGA health teams. Because the ward level data validation was conducted by facilities at similar levels and in similar geographical area, the participating health facilities staff were able to compare their performance and arrive at suitable and appropriate solutions to commonly identified problems.

The production of the State Malaria Bulletins (quarterly and annual) showing relevant performance indicators and other state-specific information on malaria provide visibility of malaria program activities to stakeholders and health authorities. This was a strategic approach to get malaria program the attention it deserves. The bulletin presents high level program performance in a clear, concise, and convincing manner to facilitate prioritizing funding for malaria program at the highest level of decision making amidst competing government priorities. The provision of State Malaria Bulletin and the display of the electronic versions on the State Ministry of Health´s website is cost saving for the MFP and ensured sustainability of the initiative. Kano and Zamfara States Ministries of Health now host the report on their websites. A culture of data use for decision making has been encouraged by the development of the DHIS2 malaria dashboard for tracking key malaria indicators. This innovative user-friendly dashboard granted the health authorities at national, state, and LGA officers´ access (unique username and password) to data visualizations for decision making at all levels. The dashboard, operationalized at higher administrative levels of health information system - LGA and the state, is an opportunity for further evaluation of the quality of data aggregated from service delivery at the health facility level. While the malaria monitoring chart provided an opportunity for data analysis at health facility level, improving accuracy of these service delivery indicators on the chart also improves the quality of aggregated data at LGA and state levels. Data-driven health facility supportive supervisory visits by state and LGA level officers based on program performance is an objective way to support health facilities and LGAs. These were designed to elicit ongoing changes in health worker´s practice and behavior over time and improve service delivery performance [8]. On-the-job training and mentoring of health facility workers by the LGA team helped build health workers´ capacity in malaria case management, data management, and use for decision making.

The project has strengthened malaria surveillance activities and addressed challenges by having accurate number of facilities as it relates to reporting rate of completeness and timeliness [5]. The MFP health facility service assessment provided updated information on health facilities in both project states and helped to assess each facility´s functionality, services provided, and data reported into the DHIS2 platform and human resource database. The updated master health facility list was given to state authorities to share with the Department of Planning, Research and Statistics (DPRS), Federal Ministry of Health to update the national list. Monthly ward level data validation meetings served as a peer-to-peer learning approach and extensive review of data for timeliness, completeness, accuracy, and reliability. The provision of access for LGA and state health officers to the malaria DHIS2 dashboard has helped in the use of data for decision making. Findings observed during the implementation of the MFP were similar to other studies that offered data quality checks and feedback, knowledge transfer activities (mentoring and training), and data review meetings and dashboards as approaches for improvement of data quality [9]. The development and innovation of the malaria DHIS2 dashboard in Kano and Zamfara States have improved data visibility, use and feedback processes for decision making at the LGA and state levels. The real-time approach of the dashboard also provides an objective approach for identifying specific health facilities for capacity building of healthcare workers to address their data quality issues and provides immediate feedback to affected HFs. Political commitment from state, LGA and Ward authorities to provide the needed resources to rectify identified gaps is very important component to improve healthcare delivery. PMI/Nigeria and NMEP have adopted the monthly ward level data validation process and implemented the strategy in the eleven PMI-supported states [10]. The NMEP has requested the MFP to support some states in their malaria program as they seek funding. The ward level data validation meetings are also being adapted by NMEP for the country´s data quality improvement strategy. The NSTOP polio program is adopting the ward level data validation strategy to improve its routine immunization data validation meetings in their supported states. The DHIS2 malaria dashboard being used in both MFP states has been well-received by the NMEP with the intention of its scale-up to other states with the support of malaria implementing partners.

## Conclusion

Innovative approaches deployed during the MFP implementation in Kano and Zamfara States have helped healthcare workers at the HF, LGA and State levels to understand, analyze, interpret, and disseminate data for decision making. Improvements in DHIS2 data quality were also observed during the project implementation. It is worthy of note that MFP´s intervention was expanded to the remaining 24 LGAs in Kano state. This was the state government´s recommendation due to the project´s impact. The overall strategy has improved malaria program performance in the project states and can be adopted to improve the efficiency of malaria program implementation in other states in Nigeria, as well as in other malaria endemic countries in Africa. Further commitment towards strengthening the capacity of health facility personnel in basic recording, reporting, analyses, interpretation, and use of data for program management will help improve the health delivery system at many levels.
